# Accessibility of and Barriers to Long-Term Follow-Up Care for Childhood Cancer Survivors

**DOI:** 10.1001/jamanetworkopen.2024.40258

**Published:** 2024-10-17

**Authors:** Jiaoyang Cai, Sara Malone, Nickhill Bhakta, Ching-Hon Pui, Jing Chen, Shaoyan Hu, Hui Jiang, Xiuli Ju, Fen Zhou, Melissa M. Hudson, Yin Ting Cheung

**Affiliations:** 1 Department of Hematology & Oncology, Shanghai Children’s Medical Center, Shanghai Jiao Tong University School of Medicine, National Children’s Medical Center (Shanghai), Shanghai, China; 2 Washington University School of Medicine, Department of Surgery, St Louis, Missouri; 3 Department of Global Pediatric Medicine, St Jude Children’s Research Hospital, Memphis, Tennessee; 4 Department of Epidemiology and Cancer Control, St Jude Children’s Research Hospital, Memphis, Tennessee; 5 Department of Oncology, St Jude Children’s Research Hospital, Memphis, Tennessee; 6 Department of Hematology & Oncology, Children’s Hospital of Soochow University, Suzhou, China; 7 Department of Hematology & Oncology, Shanghai Children’s Hospital, Shanghai, China; 8 Department of Pediatrics, Qilu Hospital of Shandong University, Jinan, China; 9 Department of Pediatrics, Union Hospital of Tongji Medical College, Huazhong University of Science and Technology, Wuhan, China; 10 School of Pharmacy, Faculty of Medicine, The Chinese University of Hong Kong, Hong Kong Special Administrative Region, China; 11 Hong Kong Hub of Paediatric Excellence, The Chinese University of Hong Kong, Hong Kong Special Administrative Region, China

## Abstract

**Importance:**

Childhood cancer survivorship programs and long-term follow-up (LTFU) practices are inadequate in most regions of China.

**Objective:**

To understand the clinician and caregiver perceptions of LTFU care and to identify barriers to adherence to LTFU care in mainland China.

**Design, Setting, and Participants:**

This survey study had a 2-phase sequential mixed-methods approach, consisting of a cross-sectional survey followed by semistructured interviews. Participants included oncology clinicians recruited through an educational seminar on LTFU and caregivers recruited through convenience sampling. Data were collected from November 2022 to September 2023.

**Main Outcomes and Measures:**

The clinician survey and interview focused on the standards and resources for LTFU care at their practicing institution and barriers to the coordination of LTFU care. For caregivers, the survey and interview focused on their awareness of and participation in LTFU care and their opinions on future LTFU care visits.

**Results:**

A total of 101 clinicians (28 [27.7%] male; 73 [72.3%] female; 46 [45.6%] aged >40 to 50 years) completed the survey (response rate: 90.2%) representing 32 institutions from 22 provinces. As for the caregivers’ survey, 164 eligible participants (36 [22.0%] male; 128 [78.0%] female) were recruited (response rate: 20.2%). The majority of the caregivers had received a high school or greater education (96 [56.7%]) and were parents of CCSs diagnosed with leukemia (67 [40.9%]), lymphoma or solid tumors (47 [28.7%]), or conditions requiring hematopoietic stem cell transplantation (50 [30.5%]). Most clinicians (74 [73.3%]) reported providing late effects care, yet only 10 (13.5%) had a dedicated follow-up clinic for CCSs. Two-thirds (64 [63.4%]) reported that the LTFU plan for each survivor is solely determined by their clinical judgment. In structured interviews, all doctors admitted to deviating from published guidelines due to challenges in implementing screening recommendations in their settings. Barriers to providing LTFU services included patient-related factors (76 [75.2%]), survivor knowledge deficits (61 [60.4%]), and the absence of dedicated LTFU clinics (61 [60.4%]). Among caregivers responding to the survey, 60 (36.6%) had never heard of late effects. Overall, 22 of 26 caregivers (84.6%) who participated in the interviews were not aware of potential late effects, although 17 (68.0%) could articulate existing conditions and symptoms that their children were experiencing.

**Conclusions:**

In this mixed-methods study involving clinicians and caregivers, substantial disparities in the uniformity and accessibility of LTFU in China were observed, suggesting the imperative need for a standardized approach to LTFU care for survivors. This includes advocating for establishment of dedicated clinics, alongside an emphasis on enhanced education and training for both clinicians and caregivers.

## Key Points

**Question:**

What are the perceptions of clinicians and caregivers regarding long-term follow-up (LTFU) care for childhood cancer survivors (CCSs) in China?

**Findings:**

In this survey study conducted among 164 caregivers and 101 clinicians, 73.3% of clinicians reported providing late-effects care, yet only 13.5% had a dedicated follow-up clinic for CCSs. Barriers to providing LTFU services included patient-related factors (75.2%), survivor knowledge deficits (60.4%), and the absence of dedicated LTFU clinics (60.4%).

**Meaning:**

These findings could contribute to establishing a national-level accreditation for LTFU programs and setting basic standards of care for enhancing survivorship services across diverse resource settings in China.

## Teaser

This survey study examines the perceptions of clinicians and caregivers regarding accessibility of and adherence to long-term follow-up care for childhood cancer survivors in China.

## Introduction

Over the past 50 years, pediatric cancer survival rates have significantly improved,^1^ resulting in a growing population of childhood cancer survivors (CCSs). By 2040, it is estimated that there will be 580 000 CCSs in the United States, with numbers expected to rise further as more children survive their disease.^2^ Similar progress in survival has been observed in China, where an estimated 40 000 new cases of childhood cancer are recorded annually.^3^ The overall reported survival rate of childhood cancer in China from 2000 to 2010 was approximately 70%, with some diagnostic groups achieving higher survival rates.^4^

Long-term follow-up (LTFU) care is important for CCSs, as their risks of morbidity and mortality increase with age.^5,6^ While no global consensus exists, several international organizations have recommended that all CCSs receive risk-based survivorship care,^7^ which includes (1) a personal surveillance plan^8^ and (2) interventions to prevent or remediate the late effects (LEs) of treatment.^9^ The Children’s Oncology Group (COG) and the International Late Effects of Childhood Cancer Guideline Harmonization Group (IGHG) provide exposure-based recommendations for the screening of LEs to support risk-based survivorship care of CCSs at least 2 years after the completion of treatment.^7,10^ Survivorship care models and programs may provide a platform for more uniform practices and implementation of such screening guidelines. The existing models described in the literature are typically classified based on type of practitioners providing care, such as physician-, nurse-, or nurse practitioner–led models. In addition, models may be led by a care team or involve shared care by multidisciplinary clinicians. A recent scoping review of care models for CCSs in Asia revealed that most institutions in China have adopted the oncology specialist care model in which oncologists provide LTFU care, and specialized LTFU programs are rare.^11^ Despite advances in the Chinese health care system, challenges such as staffing limitations, geographical diversity, and cultural differences have led to disparities in survivorship care.^12^

The goal of this study was to characterize the accessibility of and barriers to LTFU care as perceived by Chinese clinicians and caregivers of CCSs. We aimed to identify barriers to and facilitators of adherence to LTFU care from both the clinicians’ and caregivers’ perspectives as well as gain insights to inform future directions for developing educational and training programs to support LTFU in mainland China.

## Methods

### Design and Setting

We conducted a 2-phase sequential mixed-methods survey study, including (1) a cross-sectional questionnaire study followed by (2) semistructured interviews of oncology clinicians and caregivers. The surveys were distributed in November 2022 for clinicians and in February 2023 for caregivers. The semi-structured interviews were conducted from July to September 2023.

The study was approved by the institutional review board of the Shanghai Children’s Medical Center. Clinicians acknowledged at the initiation of the survey that completion and submission of the survey implied their informed consent to participate. Written or electronic informed consent was obtained from all caregiver participants. There were no incentives provided for participation in this study. The American Association for Public Opinion Research (AAPOR) reporting guideline was adopted to report the survey findings.^13^

#### Phase 1: Scoping Survey

##### Clinician Survey

We surveyed clinicians (pediatric oncologists, radiation oncologists, surgeons, and nurses) who were involved in the care of children with malignant hematologic and oncologic diseases in mainland China. The clinicians were recruited through a hybrid (onsite and online) educational seminar on the LTFU of CCSs. Primary care clinicians were not targeted in this study because they play a limited role in the care of cancer survivors in China.^11,14^

The questions included in the clinician survey were adapted from those in reports published by other pediatric oncology groups in the United States and Europe.^15,16,17,18^ The survey included 36 questions pertaining to the (1) characteristics of the clinician’s practicing institution, (2) standards and practices for the provision of care for LEs, (3) availability of resources, (4) perceptions of barriers to LTFU care, and (5) perceptions of barriers to the transition from acute oncology care to LTFU care (eTable 1 in Supplement 1).

##### Caregiver Survey

Caregivers were recruited through convenience sampling. The survey was distributed to caregivers through a survivors’ WeChat (a popular social media platform in mainland China) group, which is a private group previously created by the Shanghai Children’s Medical Center and has engaged 812 families of CCSs. To be eligible for the survey, the caregiver of the CCS had to identify themselves as a parent of a child who was at least 5 years from cancer diagnosis and currently in remission at least 2 years after completion of therapy.

The caregiver survey was adapted from other published studies on survivors’ or caregivers’ perceptions of LTFU services and awareness of LEs.^19,20^ The survey included 30 questions to assess the caregivers’ (1) sociodemographic information; (2) participation in LTFU care (referred to as off-therapy clinic) within the past 2 years and, if relevant, reasons for missing appointments; (3) awareness of cancer diagnosis and whether such information was disclosed to the child; and (4) experience and perceptions of LEs (eTable 1 in Supplement 1).

##### Survey Administration

Both the clinicians’ and caregivers’ surveys were distributed via a social media platform and autocollected on Wenjuanxing (Questionnaire Star), a widely accepted and encrypted online platform used in China for survey data collection.^21^ All responses were recorded anonymously and retrieved electronically. The survey was self-administered in Simplified Chinese and took approximately 10 to 15 minutes to complete.

#### Phase 2: Qualitative Interviews

The data obtained from the quantitative study was used to construct semistructured individual interviews (approximately 20 minutes each) aimed at further exploring clinician and caregiver perceptions of how LTFU care is currently implemented and enhancing understanding of their needs for LTFU care. The interviewers’ guide (eTable 2 in Supplement 1) was structured according to the Conceptual Framework for the Implementation of Research, which ensures that multilevel domains (inner setting, outer setting, individual, and process) are considered when discussing the determinants of LTFU care.^22^ In short, the clinicians’ interviews focused on their experiences and awareness of LTFU care and their perceptions of the coordination of LTFU care. For caregivers, the interview focused on their experiences and awareness of LTFU care and their opinions on future LTFU care visits. Potential interview participants were randomly selected following the questionnaire phase. The recordings were transcribed verbatim in Mandarin, then translated into English by 2 bilingual investigators (J.C. and Y.T.C.) for analysis.

### Statistical Analysis

Descriptive data were used to summarize the participants’ characteristics and responses. For the caregivers’ survey, logistic regression was used to identify factors associated with missing LTFU appointments and revealing cancer diagnosis to the child. The factors of interest were selected according to previous local studies that examined barriers to LTFU care and disclosure of cancer diagnosis to the child.^11,15,19,23^ We hypothesized that longer time since completion of treatment, nonreceipt of hematopoietic stem cell transplantation, and lower socioeconomic status would be associated with discontinuation LTFU care or not informing the child of their previous cancer diagnosis. Significance testing was performed using a 2-sided test, with *P* < .05 as the significance level. All statistical computing was done using R statistical software version 4.1.3 (R Project for Statistical Computing).

To analyze the qualitative data, 2 investigators independently read and coded all the transcripts, and coding was performed in 2 cycles. Inductive coding was utilized for this analysis. The coding and themes were discussed by the research team, and a coding framework was developed and applied to all the transcripts.

## Results

### Clinician Study

Of the 112 surveys distributed to clinicians, 101 returned their surveys (28 [27.7%] male; 73 [72.3%] female; 46 [45.6%] aged >40 to 50 years), yielding a 90.2% response rate. The participants represented 36 institutions from 22 provinces (eTable 3 in Supplement 1). Most respondents were pediatric oncologists (58 [57.4%]), followed by pediatric hematologists (26 [25.7%]). Most respondents were from the eastern regions of China (58 [57.4%]). Among the 101 clinicians who completed the survey, 11 (10.9%) were randomly selected to participate in the interviews, all of whom attended and completed the interviews.

#### Theme 1: Variations in the Practice and Delivery of LTFU Care

Most clinicians (74 [73.3%]), representing 31 of the 36 institutions, reported providing LTFU care. Among these 74 clinicians, approximately one-third (26 [35.1%]) followed up survivors in the treating pediatric oncology clinics, while half (38 [51.4%]) reported that their survivors were followed up via telephone or the social media platform by other non–pediatric oncology specialists, in addition to the treating pediatric oncology clinic. Only 10 respondents (13.5%), representing 5 institutions, had a dedicated LTFU clinic for CCSs, of which 4 were located in the economically developed eastern regions and 1 was in a central region.

Among the 74 clinicians who provided LTFU care, 50 (67.6%) routinely provided pretreatment counseling on LEs of treatment for patients and their families. However, only 6 (8.1%), representing 3 institutions, ensured that survivors received a personal cancer treatment summary. While general health screening (diabetes, hypertension, and hormonal disorders) was routinely performed by most (57 [77.0%]), screening for educational needs (14 [18.9%]), social or cognitive impairment (11 [14.9%]), and psychological distress (15 [20.3%]) was less common. Health promotion topics, such as weight management (69 [93.2%]), nutrition (72 [97.2%]), and physical activity (71 [95.9%]), were more uniformly discussed during LTFU. As reported by the clinicians, the LTFU services team of their practicing institutions comprised oncologists (94 [93.1%]), nurses (59 [58.4%]), social workers (39 [38.6%]), dietitians (27 [26.7%]), psychologists (20 [19.8%]), and general practitioners (10 [9.9%]).

Regarding the transition from acute care to LTFU care, approximately half of clinicians (60 [59.4%]) reported that their institutions did not have an infrastructure to match patients with adult health care practitioners. Many reported lacking a system to gather feedback (70 [69.3%]) or were uncertain (35 [34.7%]) about the transition process (Table 1).

**Table 1.** Barriers to Providing LTFU Services Endorsed by Oncology Clinicians

Barriers to providing LTFU services	Clinicians, No. (%) (N = 101)
Lack of transition and sustainable infrastructure for LTFU program	
Lack of process to match and communicate with adult health care practitioners	60 (59.4)
Lack of a system to obtain feedback from young adults about care transitions	70 (69.3)
Uncertainty about the transition process	35 (37.4)
Lack of familiarity with LTFU practices and guidelines	
Limited clinical knowledge	64 (63.9)
Unsure which guidelines to use	14 (13.9)
Lack of resources	
No financial support for the LTFU program operations in the last 5 y	82 (81.2)
No philanthropic funding for salaries in the last 5 y	85 (84.2)
No database to track survivor health outcomes	61 (60.4)
Patient-related barriers	
Patient-related factors (eg, geographical accessibility and cost)	76 (75.2)
Survivor knowledge deficits about the importance of LTFU care	61 (60.4)
Barriers to transitioning to adult care LTFU practitioners	
Survivors’ preference to be cared for by their pediatric oncologists	85 (84.2)
Not recognizing the importance of transitional care	62 (61.4)
Lack of dedicated LTFU clinics	61 (60.4)

Abbreviation: LTFU, long-term follow-up.

Most clinicians who attended the structured interviews acknowledged the importance of LTFU services for managing survivors’ physical and psychosocial effects as well as their reintegration into school (Table 2). However, clinicians endorsed variable understanding of LTFU care and services, as they seemed to have difficulty differentiating an LTFU clinic (a specialized clinic) from a treating pediatric oncology unit that provides care for survivors (in addition to acute care services). Consistent with the results of the survey, almost all clinicians reported not knowing what a treatment summary looks like or how to prepare one.

**Table 2.** Major Themes and Selected Quotations From the Clinician Interviews

Theme	Representative quotation (participant No.)
Late effects care not a common practice across institutions	“Our focus is on children who survived within 5 y of cancer diagnosis… Seldom go beyond that.” (5) “Yes, we have a After Completion of Therapy Clinic. I know late effects care is important, especially in [monitoring] their physical and psychosocial development. Parents are always very concerned about their height [growth] problems and brain [cognitive] function. Having them come back regularly for LTFU may help them reintegrate back into school and normal life.” (3) “A group of doctors are assigned to see [follow-up with] survivors as and when they are available to do so… No, it is not a single [dedicated] team. We take turns.” (5) “Yes, we have nurses from philanthropic funds to conduct surveys for survivors, and then we invite them back for follow-up.” (9) “A treatment summary… what do you mean? You mean patients get a copy of [the treatment] they received? No… A printout from our [electronic health] system? No…” (5)
Variations in the delivery of LTFU care	“Survivors are followed-up in the same oncology clinic [as they were previously treated]. Not enough manpower for a separate clinic.” (4) “We provide late effects care… no specific clinic because no dedicated team. But we try to assign the same [treating] team of doctors to see the survivor. This is considered LTFU clinic?” (3) “Not sure if this can be considered a ‘structure’ [framework] but we have initiated a system to contact survivors and invite them to come back for late effect care. First, we arrange survivors based on their date of cancer diagnosis, then contact the survivors in batches. Then we will decide whether to bring them back to hospital or to just do phone services or surveys… Follow-ups over the phone may be more appropriate as they [parents and children] are busy. It may be a good starting point if we have the manpower.” (1) “We discussed potential late effects at cancer diagnosis but by the time they finished treatment, they would have forgotten most of it… We refer them to the Sunflower CureKids Foundation [a local non-governmental organization] if they want more information about late effects.” (6) “I have visited the After Completion of Therapy Clinic at St Jude [Children’s Research Hospital], it really opens my eyes to see how survivors should be assessed and followed up. Such visits to other places [with existing LFTU infrastructures] are important. Otherwise, we wouldn’t know what models are suitable for our setting.” (8)
Lack of sustainable infrastructure for the transition to LTFU programs	“We have collaborations with an institution in Canada so our doctors are aware of the need for a transition program.… However, we need a good database documenting the patients’ treatment history to facilitate transition. Currently, we don’t have such database or application for the transfer of data.” (5) “There is an age limit set by our institutions. Once our patients turn 18 years old, we cannot keep them in our clinics anymore. We lose them when we don’t have a proper transition.” (8) “The institution needs to improve its workflow to help us contact the survivors and transit them properly… provide us with training, give us a specific time and [physical] clinic to set up LTFU services, give us resources to conduct screening… basically, this is a huge task!” (9) “Transition program? You mean from child to adult care? Not at all, we do not have any infrastructure to do that. Patients just move on. At most we would give them some guidance but they are supposed to fix their follow-up appointments with other specialists themselves.” (7)
Challenges in implementing recommended practices or guidelines	“Yes, I am aware of the most famous [authoritative] guideline, the COG one. But I still use my own discretion to decide how the survivor should be treated [followed]…. Well I agree that this is not a good practice because different doctors have different years of experience.” (3) “No, our institution does not follow the guidelines. Those guidelines are from other countries. Not that practical in my province. I know they are good but we cannot follow them due to logistic reasons.” (2) “Yes, we should follow those guidelines. But how are we going to set up so may referral services to cardiologists, endocrinologists, and other specialists? It gets even more difficult when they become adults. We need a multidisciplinary team but there isn’t one now.” (5)
Barriers from institutions’, clinicians’, and patients’ perspectives	“The institution will not prioritize resources for LTFU because there are other more immediate needs from the hospital’s perspective…. They don’t see the importance of putting resources on survivors who are supposed to be well. We may need to start everything from scratch on our own first. And it is difficult to start a clinic without a dedicated team or manpower support from the management.” (3) “Our doctors are starting to appreciate the importance of late effects care. But no time and resources to do that.” (2) “We only rely on online resources to learn more about late effects care… you know, those from COG. More concrete and dedicated training on rehabilitation is needed…. To do this, we need time-off from clinical work. We need a lot more help and effort to implement these guidelines into practice.” (4) “Doctors tell them [the patients] that they are well after completing treatment—they meant that at that point in time, the cancer or tumor is treated and is gone. But the parents and patients misunderstand that they are ‘well’, and that they do not need to come back for follow-up ever again. Hence, education and communication are important.” (11) “Sorry for being [rude]—but the patients, especially parents, themselves are a huge barrier! They don’t want to be contacted again after completing treatment. They even changed their names and contact numbers.” (8) “The children and parents are busy. They don’t want to travel all the way back to the clinic. They said, ‘Missing school is a big [unacceptable] thing for my child!’. How to explain to them that LTFU is important, and that they need to come back for follow-up?” (6) “[The parents’] perspectives need to change. Some of them do not want to let their child know about their previous cancer diagnosis. So, they want to cut ties with the hospital immediately after completing treatment.” (9)

Abbreviations: COG, Children’s Oncology Group; LTFU, long-term follow-up.

#### Theme 2: Familiarity With LTFU guidelines

Regarding familiarity with LTFU guidelines, two-thirds reported that the LTFU plan for each survivor was solely based on their clinical judgment (64 [63.4%]) or they were unsure of which guidelines to use (14 [13.9%]). Only a minority used COG (18 [17.8%]), IGHG (3 [3.0%]), or in-house (5 [5.0%]) guidelines.

During the semistructured interviews (Table 2), all clinicians reported lack of adherence to published guidelines and a preference to use their own clinical discretion. Reasons endorsed for this preference included the inability to effectively implement guidelines, insufficient referral systems and resources, or perceptions that certain screening tests were not important for asymptomatic survivors.

#### Theme 3: Barriers From the Institution, Clinician, and CCS or Caregiver Perspectives

The top 3 barriers to providing LTFU services endorsed by clinicians included patient-related factors (eg, geographic accessibility and cost) (76 [75.2%]), lack of survivor knowledge about the importance of LTFU care (61 [60.4%]), and a lack of dedicated LTFU clinics (61 [60.4%]) (Table 1). In terms of financial barriers, 82 clinicians (81.2%), representing 28 institutions, reported that there was no funding support for LTFU program. Specific to institutional barriers, 61 (60.4%) reported the lack of a database to track survivors’ health outcomes.

Only 1 clinician interviewed had access to an existing specialized team for LTFU care. In the other clinicians’ institutions, there were no specific oncologists appointed to provide LTFU care because of the competing demands of patients requiring acute care. They also acknowledged their lack of experience and training in rehabilitation (Table 2).

### Caregiver Study

eTable 4 in Supplement 1 presents the characteristics of the CCSs and caregivers. Of the 812 eligible CCSs, 164 of their caregivers (36 [22.0%] male; 128 [78.0%] female) responded to the request to enroll (20.2% response rate). The median (range) current age of CCSs was 9.9 (4.6-21.7) years. Most CCSs were diagnosed with hematological malignancies (67 [40.9%]) or non–central nervous system solid tumors (47 [28.7%]). A substantial proportion (142 [86.6%]) reported that their child’s health care was covered by medical insurance, with 6 having commercial insurance. Of the 164 respondents who completed the survey, 26 were randomly selected to participate in the interviews, and all completed their interviews.

#### Theme 1: Conflicting Perceptions of the Importance of LTFU Services

Of the 164 caregivers, 102 (62.2%) reported attending at least 1 off-therapy clinic appointment during the past 2 years, while the remaining 62 (37.8%) reported missing 1 or more LTFU appointments. Sixteen caregivers (25.8%) reported COVID-19 quarantine restrictions as the primary reason for missing LTFU appointments. For the remaining 48 caregivers, the most commonly reported non–COVID-19–related reasons for missing LTFU appointments were reluctance to travel (14 [29.2%]), the child feeling well (10 [20.8%]), and not wanting the child to miss school (7 [14.6%]). The multivariable analysis showed that more than 5 years since completing therapy was the only significant risk factor associated with missing an LTFU appointment in the past 2 years (odds ratio [OR], 3.9; 95% CI, 2.0-8.3; *P* < .001) (Table 3). There was no association of previous treatment with hematopoietic stem cell transplantation and socioeconomic factors with missing LTFU appointments.

**Table 3.** Factors Associated With Missing LTFU Care and Not Revealing Cancer Diagnosis to Child Endorsed By Caregivers

Survivor and caregiver characteristics	Total	Missing LTFU			Not revealing cancer diagnosis to child		
		Missed LTFU, No. (%)	OR (95% CI)	*P* value	Not revealing diagnosis to child, No. (%)	OR (95% CI)	*P* value
Child’s age at the time of interview, y^a^	164	NA	1.0 (0.9-1.1)	.92	NA	1.5 (1.3-1.8)	<.001
Years after therapy							
2-5 y	81	18 (22.2)	1 [Reference]	<.001	36 (44.4)	1 [Reference]	.005
>5 y	83	44 (53.0)	3.9 (2.0-8.3)		49 (59.0)	3.2 (1.4-7.2)	
Hematopoietic stem cell transplantation							
No	114	44 (38.6)	1 [Reference]	.91	65 (67.0)	1 [Reference]	.08
Yes	50	18 (36.0)	1.0 (0.5-2.2)		20 (40.0)	0.5 (0.2-1.1)	
Caregiver level of education							
High school or below	96	33 (34.4)	1 [Reference]	.36	46 (47.9)	1 [Reference]	.45
University degree or above	68	29 (42.6)	1.4 (0.7-3.1)		39 (57.4)	0.7 (0.3-1.7)	
Residence							
Urban	112	47 (42.0)	1 [Reference]	.54	63 (56.3)	1 [Reference]	.08
Rural	52	15 (28.8)	0.8 (0.3-1.8)		22 (42.3)	0.5 (0.2-1.1)	
Insurance							
Yes	142	55 (38.7)	1 [Reference]	.58	74 (52.1)	1 [Reference]	.38
No	22	7 (31.8)	0.7 (0.2-2.1)		11 (50.0)	0.6 (0.2-1.9)	
Annual income of the household, ¥^b^					77 (52.4)		
<250 000	147	55 (37.4)	1 [Reference]	.68	8 (47.1)	1 [Reference]	.50
>250 000	17	7 (41.2)	0.8 (0.2-2.5)		77 (52.4)	0.6 (0.6-2.4)	

Abbreviations: HSCT, hematopoietic stem cell transplantation; LTFU, long-term follow-up; NA, not applicable; OR, odds ratio.

^a^ The child’s age at the time of interview is analyzed as a continuous variable (years) in the multivariable model.

^b^ To convert Chinese yen to US dollars, multiply by 0.138.

In the interviews, half of the caregivers (13 [50.0%]) recognized the importance of LTFU care for detecting relapse and secondary malignant neoplasms but perceived them to be less valuable for preventing or screening for LEs (Table 4). Six caregivers (23.1%) explicitly mentioned that they had no intention of participating in LTFU services (even if they were to be conducted in the same residential city) because they felt that their children were healthy.

**Table 4.** Major Themes and Selected Quotations From the Caregiver Interviews

Theme	Representation quotation (Participant No., relationship to patient)
Conflicting perceptions on the importance of LTFU services	“If I can overcome transportation challenges and financial problems, then coming back to the hospital [for LTFU] once a year is acceptable, I guess.” (8, father of a survivor of neuroblastoma) “I think having a doctor to follow up on my child is important, especially her growth and developmental problems, and fertility issues next time when she grows up because she had radiation on her abdominal area.” (11, mother of a survivor of Wilms tumor) “Late effects care is important, I mean, the child needs to be well holistically.” (18, mother of a survivor of rhabdomyosarcoma) “I am open to bringing my child back for follow-up. But maybe once every 2 years instead of once every year? It is difficult to make an appointment and arrange for the logistics.” (3, father of a survivor of neuroblastoma) “No, I don’t think I want to return to Shanghai [the hospital where the child received primary care]. I prefer to see a local doctor in my province instead.” (12, mother of a survivor of Wiskott Aldrich syndrome after transplant) “She [the child] is well and even the doctor said that she has recovered. I don’t think there is a need for follow-up.” (22, mother of a survivor of juvenile granular monocytic leukemia after transplant) “We live too far away from the hospital that he received cancer treatment. My child needs to go to school. We don’t want him to miss school.” (24, mother of a survivor of Langerhans cell histiocytosis)
Lack of awareness of treatment-related late effects	“Late effects? No… I am more concerned about the cancer relapse.” (19, mother of a survivor of Langerhans cell histiocytosis) “Not too sure about late effects from the treatment…. But my child has vision problems and we know it is likely due to the tumor pressing against his eye.” (21, mother of a survivor of yolk sac tumor) “My child did not have any side effects [late effects] from treatment. The doctors did not provide much information about it.” (4, mother of a survivor of diffuse large B cell lymphoma) “My child had bad experience from the [active] treatment period. He doesn’t want to talk about it [treatment and late effects] and is depressed. Is this considered a late effect too?” (14, mother of a survivor of myelodysplastic syndrome after transplant)
Truth-telling and cultural barriers to transition experienced by caregivers and survivors	“I mean, I am okay with letting my child know about the cancer diagnosis. But I need some form of emotional support for them too.” (1, father of a survivor of neuroblastoma) “I don’t want people to know about it [the child’s cancer history]. Only his teacher knows about it. Even if I need to take time off from school to bring him to the clinic, I will not let his classmates know that he has health problems.” (2, mother of a survivor of rhabdomyosarcoma) “Everything is fine now, I don’t need my child to know about how sick he was. I prefer it that way.” (3, father of a survivor of neuroblastoma) “He doesn’t know. Even if he needs to know about it, I don’t know how to explain to him [about the cancer and treatment].” – (2, mother of a survivor of rhabdomyosarcoma) “I have no choice now that he has older…. He looked for information about cancer himself using the internet. I am worried that he will get misinformation from the internet. We have to tell him sooner or later that he had cancer when he was young.” (9, mother of a survivor of acute lymphocytic leukemia)

Abbreviation: LTFU, long-term follow-up.

#### Theme 2: Lack of Awareness of Treatment-Related LEs

Of 164 surveyed caregivers, 60 (36.6%) stated they had never heard of LEs, and none had received a treatment summary. When asked about specific LEs about which they were most concerned, caregivers endorsed concerns regarding growth problems (122 [74.4%]), secondary cancers (99 [60.4%]), and fertility (78 [47.6%]); however, only 84 (51.2%), 51 (30.1%), and 60 (36.5%) were informed about their risk of the respective LEs by their doctors (Figure).

**Figure.**
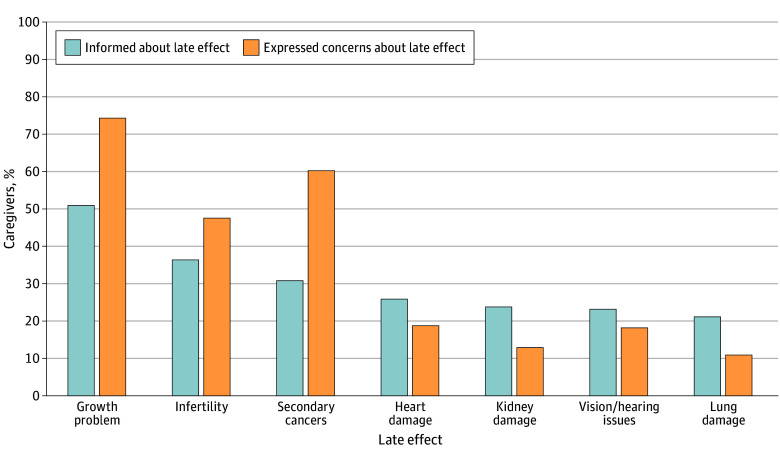
Proportion of Caregivers Who Were Concerned About vs Who Were Actually Informed About Specific Late Effects

Most caregivers (155 [94.5%]) were willing to seek information about LEs. Their preferred methods were through internet websites (122 [74.4%]), brochures (65 [39.6%]), public lectures (56 [34.1%]), hospital educational sessions (54 [32.9%]), and parent groups (46 [28.1%]).

Almost all interviewed caregivers mentioned that they were not aware of potential treatment-related LEs, although many (17 of 26 [68.0%]) could articulate some existing conditions and symptoms that their children were experiencing. Many expressed during the interview that they had not been given enough information by doctors on what LTFU care entails (Table 4).

#### Theme 3: Truth-Telling and Cultural Barriers to LTFU Transition Experienced by Caregivers and Survivors

All caregivers were aware of their child’s cancer diagnosis. However, only half (85 [51.8%]) reported revealing their cancer diagnoses to the CCS in their care. The multivariable analysis showed that a younger age of the child at the time of interview (OR, 1.5; 95% CI, 1.3-1.8; *P* < .001) and completing therapy more than 5 years ago (OR, 3.2; 95% CI, 1.4–7.2; *P* = .005) were associated with an unwillingness to disclose the cancer diagnosis to the child (Table 3).

During the interview (Table 4), caregivers expressed concerns about their child being stigmatized by their peers. Some expressed that they did not want to be reminded of the treatment experience, or they felt that it was unnecessary for the child to be aware of the diagnosis. Most caregivers perceived school commitments as a competing demand to receiving LTFU care (Table 4).

## Discussion

To our knowledge, this is the first and largest study to characterize the accessibility of LTFU care for CCSs in mainland China and identify barriers perceived by clinicians and CCS caregivers. The data illuminate a landscape in which LTFU care across China is inconsistently provided, largely in treating pediatric oncology clinics and typically without a standardized risk-based approach. Our survey results reveal significant disparities in the uniformity and accessibility of follow-up care, with specialized LTFU clinics existing in only 5 institutions, of which 4 were located in high socioeconomic cities of eastern China. This geographical imbalance of institutions with existing LTFU clinics underscores a critical need for better resource allocation and the development of guidelines and local infrastructure tailored to the varying economic capacities of regions across the country.^24^ Our data emphasize the urgent need to establish a national-level accreditation for LTFU programs and basic standards for survivorship services across the diverse resource settings in China.

In our study, 60% to 70% of clinicians reported having no infrastructure in place to communicate with adult health care practitioners or obtain feedback from clinicians and survivors on the transition process. The findings highlight a critical gap in transition planning and a lack of sustainable infrastructure for LTFU programs. Similar deficits have been reported by COG institutions that lack processes for matching survivors and communicating with adult care practitioners during care transitions.^15,16^ The results collectively underscore a pressing need for a sustainable model that facilitates the transition from pediatric to adult care. Encouragingly, the Chinese government has been active in implementing many policies to promote health care access over recent years. For example, the current national cancer control plan in China focuses heavily on preventing cancer and reducing disparities in cancer treatment.^25^

Our findings also highlight a substantial knowledge deficit among both clinicians and caregivers regarding LEs and the importance of LTFU care as well as a lack of access to resources, such as treatment summaries, that are used to facilitate care. This calls for the provision of patient navigation and advocacy, especially targeting caregivers from rural settings or with poor health literacy, to improve their understanding and engagement with LTFU services.^26^ Notably, a significant number of caregivers expressed geographical accessibility as a barrier to LTFU services, emphasizing the importance of virtual health care to coordinate the transition from acute care to LTFU care. A scoping review study that evaluated virtual care models of cancer survivorship identified increasing interest in using technology to help support survivors.^27^ Telehealth services that are culturally adapted could offer viable solutions and bridge the gaps in accessibility to overcome the geographic barrier experienced by CCS in China.

The cultural reluctance to discuss prognosis or adverse outcomes in China poses a significant barrier to effective LTFU care. Asian families often prefer clinicians to consult them before disclosing bad news or true diagnoses to a related patient, with families sometimes choosing to withhold information.^28^ This was highlighted in the interviews in our study, in which caregivers discussed a lack of disclosure with their children around their diagnosis. As noted in the narrative review by Mori et al,^28^ cultural nuances require the integration of communication skills training for health care practitioners in Asia to help them navigate the delicate balance between truth-telling and respecting patient and family preferences. The concept of “full-cycle, whole family, whole-person rehabilitation”^14^ should form the pillars of survivorship care for Chinese children with cancer and ideally involve a child-life specialist to prepare the child psychologically before disclosing health information. Patient-reported outcomes can offer a more comprehensive understanding of the child’s perspectives of their condition and lead to an enhanced quality of care while using communication strategies that are both culturally sensitive and patient-centered.^29^

### Limitations

This study has several limitations. Despite adopting a purposive sampling approach to ensure that clinicians from as many institutions as possible were recruited, respondents from certain geographical regions, especially rural areas, were difficult to recruit, as clinicians from lower resource settings tend to have less communication with professional societies. In the caregivers’ survey, sampling bias could not be avoided because of our convenience sampling approach. Moreover, the response rate of caregivers was low, and they were recruited from a single institution. However, the low participation rate might also reflect caregivers’ poor awareness of survivorship and LTFU care, and their lack of understanding of its importance. This underscores the challenges in engaging caregivers on this topic. Furthermore, as demonstrated by the clinicians’ survey, the provision of LTFU care varies across the institutions and such differences may lead to difficulty in interpreting caregivers’ experiences with LTFU care in a multicentered study. While the external validity of the study findings should be interpreted with caution, future studies should focus on conducting in-depth needs assessments of each city and/or province and identifying strategies to increase caregiver engagement and participation. Despite these limitations, this study was the first we are aware of that provides preliminary insights into the accessibility of LTFU care in China at a national level.

## Conclusions

This survey study observed substantial disparities in the provision of LTFU to CCSs. These results call for a concerted effort to improve education on the importance of LTFU care for both health care practitioners and patients’ families in China. Pertinent recommendations for future directions include developing and implementing national guidelines for LTFU care that can be adapted to local resource levels and fostering a multidisciplinary approach that includes a dedicated budget and infrastructure for LTFU programs. The insights gained from this study can serve as a catalyst for policy changes and the development of a more cohesive, equitable, and effective LTFU care framework in China.

## Notes

Supplement 1. **eTable 1.** Questionnaire and Response Options **eTable 2.** Semistructured Interview Guide **eTable 3.** Clinicians’ Institutions and Response Rates **eTable 4.** Demographic and Characters of Caregiver Respondents and Survivors

Supplement 2.
Data Sharing Statement
