# Supplementary material for: Accessibility of and Barriers to Long-Term Follow-Up Care for Childhood Cancer Survivors
Source: JAMA Netw Open. 2024 Oct 17;7(10):e2440258. doi: 10.1001/jamanetworkopen.2024.40258 (PMC11581527; doi:10.1001/jamanetworkopen.2024.40258)
Supplement: Supplement 1. — eTable 1. Questionnaire and Response Options eTable 2. Semistructured Interview Guide eTable 3. Clinicians’ Institutions and Response Rates eTable 4. Demographic and Characters of Caregiver Respondents and Survivors [file jamanetwopen-e2440258-s001.pdf]

Supplemental Online Content

Cai J, Malone S, Bhakta N, et al. Accessibility of and barriers to long-term follow-up care for childhood cancer survivors. *JAMA Netw Open.* 2024;7(10):e2440258.
doi:10.1001/jamanetworkopen.2024.40258

eTable 1. Questionnaire and Response Options

eTable 2. Semistructured Interview Guide

eTable 3. Clinicians' Institutions and Response Rates

eTable 4. Demographic and Characters of Caregiver Respondents and Survivors

This supplemental material has been provided by the authors to give readers additional information about their work.

© 2024 Cai J et al. *JAMA Network Open.*

eTable 1. Questionnaire and Response Options

	Clinicians' survey	
**Section 1: Demographic information**		
	**Questions**	**Options**
1	Sex	• Male<br>• Female
2	Age group	• 20-29 years<br>• 30-39 years<br>• 40-49 years<br>• 50-59 years<br>• 60-69 years<br>• 70 plus years
3	Name of the institution	Free text
4	Specialty	• Oncologist<br>• Radiation oncologist<br>• Surgeon<br>• Nurse
5	Age group trained to treat for cancer	• Children (≤ 18 years old)<br>• Adults (>18 years old)<br>• Both
6	Type of institution	• General hospital<br>• Specialized hospital (e.g., hematological centers, cancer centers)<br>• General pediatric/children hospital<br>• Others
7	The upper age limit for newly diagnosed pediatric oncology patients to begin treatment at your institution	• 14 years<br>• 18 years<br>• Others
8	Average number of new pediatric oncology patients treated at your institution annually	• < 50<br>• 51–100<br>• 101-150<br>• 151-200<br>• 201-250<br>• 251-300<br>• 301-350<br>• 351-400<br>• 401-450<br>• 451-500<br>• > 500
**Section 2: Standards and practices for the provision of care for late effects**		
9	Does your institution provide late effects care for CCSs?	• Yes<br>• No<br>• Not sure
10	Which model best describes pediatric cancer survivorship care in your institution?	• No specialized LTFU provided<br>• In a regular pediatric oncology clinic by health care professional staffing clinic or by CCS's treating oncologist<br>• During a regular pediatric oncology clinic, and having follow-up physician or nurse followed up by phone or WeChat

© 2024 Cai J et al. *JAMA Network Open.*

		• In a specialized LTFU program by designated providers in pediatric institution<br>• In a Specialized LTFU Program by designated providers in adult institution<br>• In a combined adult/pediatric specialized LTFU clinic<br>• Combination of specialized LTFU clinic and local primary care providers<br>• In the process of establishing a model<br>• Others
11	What are the eligibility criteria for entry into LTFU services?	• At least 2 years off treatment<br>• At least 3 years off treatment<br>• At least 5 years off treatment<br>• At least 5 years from diagnosis<br>• Time from diagnosis: specify years<br>• Time from completion of therapy: specify years<br>• Others
12	Are self-referral for LTFU services allowed?	• Self-referral is accepted<br>• Physician referral is necessary
13	What is the percentage of pediatric oncology patients who are eligible for a survivorship visit were seen in a late effects or off-therapy clinic at your institution over the past year?	• Less than 25%<br>• 25-50%<br>• 50-75%<br>• Greater than 75%<br>• Not sure
14	What is the frequency of LTFU appointments offered in your institution?	• One day/week<br>• 2–3 days/week<br>• 4–5 days/week<br>• 1 day per month<br>• 2 days per month<br>• Others<br>• Not sure
15	Do CCSs receive a copy of their cancer treatment summary?	• Yes<br>• No<br>• Not sure
16	When are the treatment summaries given?	• At end of therapy<br>• During LTFU clinic visits<br>• Both<br>• Others<br>• Not sure
17	CCSs receive a detailed written account of their visit	• Yes<br>• No<br>• Not sure
18	Do you routinely perform pretreatment counselling regarding adverse/late effects of treatment, especially fertility?	• Yes<br>• No<br>• Not sure
19	Are fertility specialists available at your institution?	• Yes<br>• No<br>• Not sure

© 2024 Cai J et al. *JAMA Network Open.*

20	Do you review vaccination status of CCSs at every LTFU visit?	• Yes<br>• No<br>• Not sure
21	Do you evaluate cardiovascular disease risk factors of CCSs who have received cardio-toxic treatments?	• Yes<br>• No<br>• Not sure
22	Do you discuss with CCSs regarding the following lifestyle factors?<br>• Keeping a healthy bodyweight<br>• Eating a healthy; balanced diet<br>• Having regular physical activity	• Yes<br>• No<br>• Not sure
23	Do you routinely perform psychosocial or psychological distress screening?	• Yes<br>• No<br>• Not sure
**Section 3: Availability of resources**		
24	Does your institution receive financial support for your LTFU program operations in the past 5 years	• Yes<br>• No<br>• Not sure
25	Does your institution receive any level of philanthropic funding for salary support for some or all personnel in the past 5 years?	• Yes<br>• No<br>• Not sure
26	Does your institution receive philanthropic support (not as a part of research funding) which enables clinical care for patients in the past 5 years?	• Yes<br>• No<br>• Not sure
27	How do you determine the type and frequency of late effects focused screening?	• Determined for each patient by clinician performing survivorship evaluation; no specific guidelines are used<br>• Determined according to institutional best practices guidelines<br>• Determined according to established survivorship guidelines<br>• Not sure
28	If guidelines are adopted, what are they?*	• Children's Oncology Group<br>• International Guideline Harmonization Group<br>• Others
29	Which types of professionals are involved in LTFU?*	• Pediatric oncologist<br>• Primary care physician<br>• Registered Nurse<br>• Nutritionist<br>• Social worker<br>• Education specialist<br>• Neuropsychologist<br>• Psychologist<br>• Physical therapist<br>• Research coordinator<br>• Trainee (fellow; resident; etc.)<br>• Others
30	Does your institution have a database that is used to track CCSs health care	• Yes<br>• No

© 2024 Cai J et al. *JAMA Network Open.*

	outcomes and/or other pertinent clinical information	• Under development<br>• Some patients only: specify conditions<br>• Not sure
**Section 4: Perceptions about barriers to LTFU care**		
31	What are the barriers in caring for CCSs?*	• Lack of process to match and communicate with adult health care providers<br>• Lack of a system to obtain feedback from young adults about care transitions<br>• Uncertainty about the transition process<br>• LTFU practices based on clinical judgment<br>• Unsure which guidelines to use<br>• Patient-related factors (geographical accessibility and cost)<br>• CCS knowledge deficits about the importance of LTFU care<br>• We do not have any barriers
32	Do you agree that health insurance/cost as a barrier to transition of survivorship care?	• Strongly agree<br>• Agree<br>• Neither agree nor disagree<br>• Disagree<br>• Strongly disagree
**Section 5: Perceptions about barriers to the transition from acute oncology care to LTFU care**		
33	Does your institution have an established process to match and communicate with CCSs' selected adult providers?	• Yes<br>• No<br>• Not sure
34	Does your institution have a mechanism to systematically obtain feedback from young adults about transition process?	• Yes<br>• No<br>• Not sure
35	How is transited CCSs' cancer related/risk-based care managed?	• Our CCSs are not transitioned elsewhere<br>• Transition to an adult-focused oncologist in an adult cancer setting<br>• Transition to a Primary Care Provider of the patient's choice<br>• Transition to a Primary Care Provider who collaborates with the pediatric survivorship/treatment team<br>• Unsure of any specific transition process<br>• Others
36	What are the barriers to the transition from acute oncology care to LTFU care?*	• CCS lack of health care insurance or insurance limitations<br>• CCS knowledge deficits about the importance of maintaining cancer related follow-up into adulthood<br>• CCSs preference to continue follow-up at the treating institution or with treating oncologist<br>• Lack of CCS access to a primary care provider for reasons other than insurance such as geography<br>• Personal preference to keep CCS in follow-up at pediatric cancer center

© 2024 Cai J et al. *JAMA Network Open.*

		• We do not have any barriers<br>• Others
	**Caregivers' survey**	
Section 1: Demographic information		
1	CCS Date of birth	Free text
2	CCS Sex	• Male<br>• Female
3	CCS type of cancer	Free text
4	CCS type of health insurance	• Government-based/social insurance<br>• Private insurance<br>• No insurance<br>• Others
5	Caregiver relationship with CCS	• Mother<br>• Father<br>• Grandparents<br>• Others
6	Caregiver level of education	• Primary school (Grade 1 - 5/6)<br>• Secondary school (Grade 7 - 9)<br>• High school (Grade 10 - 12)<br>• Completed high school<br>• Bachelor's degree<br>• Master or above
7	Caregiver employment status	• Full-time student<br>• Full-time employment<br>• Part-time employment<br>• Housewife<br>• Unemployed<br>• Retired
8	Residence	• Rural<br>• Urban
9	Annual household income	• <￥50,000<br>• ￥50,001-￥100,000<br>• ￥100.001-￥150,000<br>• ￥150.001-￥250,000<br>• >￥250,000
**Section 2: Participation in LTFU care**		
10	Did you receive any check-up at the hospital in last 2 years where their cancer was treated (please indicate month and year of your last visit)	• Yes<br>• No<br>• Not sure
11	If you have missed or discontinued LTFU care, what are reasons?*	• Because the doctor said my child did not need to go anymore<br>• Because I moved to another city or town<br>• Because of the costs of missing work or traveling<br>• Because of need to care for other children in the family<br>• Because my child is doing well after stopping treatment<br>• Because I don't want my child to miss school

© 2024 Cai J et al. *JAMA Network Open.*

		• Because I do not want to go for other reasons<br>• COVID restrictions or home qurantine<br>• Others
12	What is your preferred mode of off-therapy follow-up visits?	• Local pediatrician /general medicine doctor/ neighborhood clinic<br>• Pediatric oncologist who treated your child<br>• Other specialist (heart specialist, hormone specialist, eye specialist, etc…)<br>• Others
13	Would you want your child to attend an off-therapy program that checks for late effects?	• Yes<br>• No<br>• Not sure
14	How long would you likely return for follow-up?	• For at least 2 years after therapy<br>• For at least 5 years after therapy<br>• Until my doctors tell me to stop follow-up<br>• Not sure<br>• Others
	Would you like to get information about late effects?	• Yes<br>• No<br>• Not sure
15	Preferred method of receiving information*	• Parent groups<br>• Public lectures<br>• Websites on the internet<br>• Books<br>• Hospitals<br>• Others
**Section 3: Awareness of cancer diagnosis and whether such information was disclosed to the child**		
16	Duration off therapy	• Between 2 and 5 years<br>• 5 or more years<br>• Not sure
17	Relapse status	• Yes<br>• No<br>• Not sure
18	Is your child aware of their diagnosis?	• Yes<br>• No
19	If your child is not aware of their diagnosis, why not?*	• My child is too young<br>• I don't want my child to know<br>• Stigma from peers<br>• Others
20	Treatment received?*	• Chemotherapy<br>• Radiotherapy<br>• Bone marrow transplantation<br>• Surgery<br>• Did not receive treatment<br>• Not sure<br>• Others
21	Radiation site(s)? *	• Brain<br>• Head and face<br>• Neck

© 2024 Cai J et al. *JAMA Network Open.*

<table>
<tr><td></td><td></td><td>• Chest
• Spine
• Abdomen
• Pelvis
• Arm
• Leg
• Total body irradiation
• Did not receive radiation
• Not sure
• Others</td></tr>
<tr><td>22</td><td>Surgery?*</td><td>• Biopsy only
• Surgery on an arm or leg (like limb-sparing or amputation)
• Chest surgery
• Abdominal surgery
• Brain surgery
• Removal of an organ (like the kidney)
• Other type of surgery
• Did not receive surgery
• Not sure</td></tr>
<tr><td>23</td><td>Bone marrow transplant?</td><td>• Using cells from their own blood or bone marrow
• Using cells from someone else’s blood or bone marrow
• Did not receive transplant
• Not sure</td></tr>
<tr><td colspan="3">Section 4: Experience of and awareness of late effects</td></tr>
<tr><td>24</td><td>Have you ever heard of the term “late effects” before?</td><td>• Yes
• No
• Not sure</td></tr>
<tr><td>25</td><td>Where did you get information about late effects?*</td><td>• My child’s doctor
• Parent groups
• Public lectures
• Websites on the internet
• Books
• I have never heard of late effects
• Others</td></tr>
<tr><td>26</td><td>Do you have a treatment summary?</td><td>• Yes
• No
• Not sure</td></tr>
<tr><td>27</td><td>Have you been told that your child might experience the following problems because of cancer treatment?*</td><td rowspan="3">• Vision/hearing problem
• Lung damage
• Problems with infections
• Increased risk of second cancers
• Low hormone levels
• Heart damage
• Kidney damage
• Difficulty getting pregnant or fathering children
• Growth problems</td></tr>
<tr><td>28</td><td>What three problems on the above list that would concern you the most?*</td></tr>
<tr><td>29</td><td>Has your child experienced health problem?*</td></tr>
</table>

© 2024 Cai J et al. *JAMA Network Open.*

		• I have never been told about risks of any of these problems<br>• Others
30	Do you think the problem could have been related to your child's cancer or treatment?	• Yes<br>• No<br>• Not sure

CCS: childhood cancer survivors; LTFU: long-term follow-up

*Respondents can select multiple options.

© 2024 Cai J et al. *JAMA Network Open.*

eTable 2. Semistructured Interview Guide

	Providers	Caregivers
**Inner setting**	1. How much do you know about LTFU care?<br>2. What is your experience in LTFU care?<br>3. Do you think that it is important to do LTFU? Why?<br>4. Do you provide detailed treatment summary and explanations to parents regarding treatment-related long-term side-effects?	1. How much do you know about LTFU care?<br>2. Do you think that it is important for your child to receive LTFU? Why?<br>3. Could you please tell me about the main symptoms/side-effects your child has experienced since completing cancer treatment?<br>4. Do you have any concerns about your child's risk for future side-effects related to the cancer and treatment?
**Outer setting**	1. What resources (i.e., staffing, training, equipment) are available within the organization to facilitate LTFU care?	1. Are there any factors (i.e., lack of medical insurance, longer distance from residence to cancer center) that may affect your willingness to let your child receive LTFU care?
**Individual**	1. What skills or knowledge do you think are necessary to effectively implement LTFU care?<br>2. Can you describe any experiences or prior training that have prepared you to implement LTFU care?	1. Has participating in LTFU had any impact on your work/ your child's school?<br>2. How do your personal beliefs and attitudes influence your willingness to let your child to participate LTFU care?
**Implementation process**	1. How do you want to implement LTFU care?<br>2. Do you have any suggestions that could help to enhance LTFU care?	1. Do you have plans to let your child participate in LTFU care? What are some barriers that might make you think twice about attending a LTFU clinic?<br>2. Do you have any suggestions that could help to enhance the LTFU care?

LTFU: long-term follow-up.
The questions were developed according to the Conceptual Framework for Implementation Research.

© 2024 Cai J et al. *JAMA Network Open.*

eTable 3. Clinicians' Institutions and Response Rates

Hospital	City	Regions	Distributed n	Responded n	Response rate (%)
Shanghai Children's Medical Center	Shanghai	Eastern	14	10	71.4
Shanghai Children's Hospital	Shanghai	Eastern	10	10	100
Children's Hospital of Fudan University	Shanghai	Eastern	2	2	100
Children's Hospital of Nanjing Medical University	Nanjing	Eastern	2	1	50
Children's Hospital of Soochow University	Suzhou	Eastern	13	7	53.8
Xuzhou Children's Hospital	Xuzhou	Eastern	2	2	100
Anhui Medical University Second Affiliated Hospital	Hefei	Central	1	1	100
Union Hospital Affiliated to Fujian Medical University	Fuzhou	Eastern	2	2	100
Guangxi Zhuang Autonomous Region People's Hospital	Nanning	Western	1	1	100
Guangzhou Women and Children's Medical Center	Guangzhou	Eastern	1	1	100
Shenzhen Children's Hospital	Shenzhen	Eastern	1	1	100
The Seventh Affiliated Hospital of Sun Yat-sen University	Shenzhen	Eastern	1	1	100
Nanfang Hospital, Southern Medical University	Guangzhou	Eastern	1	1	100
Affiliated Hospital of Guizhou Medical University	Guizhou	Western	2	2	100
Zunyi Medical University Affiliated Hospital	Zunyi	Western	1	1	100
Hainan Women and Children's Medical Center	Haikou	Eastern	3	3	100
Hebei Children's Hospital	Shijiazhuang	Eastern	1	1	100
Hunan Children's Hospital	Changsha	Central	1	1	100
The Third Xiangya Hospital	Changsha	Central	2	2	100
Xiangya Hospital Central South University	Changsha	Central	1	1	100
Union Hospital of Tongji Medical College, Huazhong University of Science and Technology	Wuhan	Central	11	11	100
Tongji Hospital of Tongji Medical College, Huazhong University of Science and Technology	Wuhan	Central	3	3	100
Wuhan Children's Hospital	Wuhan	Central	4	4	100
First Hospital of Jilin University	Changchun	Northeast	1	1	100
Jiangxi Provincial Children's Hospital	Nanchang	Central	1	1	100
Qilu Hospital of Shandong University	Jinan	Eastern	7	7	100
Affiliated Hospital of Qingdao University	Qingdao	Eastern	1	1	100
Shandong tumor hospital	Jinan	Eastern	1	1	100
Qinghai Women and Children's Hospital	Xining	Western	1	1	100
West China Second University Hospital	Chengdu	Western	3	3	100
The Second Affiliated Hospital of Wenzhou Medical University	Wenzhou	Eastern	1	1	100
Xi'an Northwest Women's and Children's Hospital,	Xi'an	Western	2	2	100
Children's Hospital of Xinjiang Uygur Autonomous Region	Wulumuqi	Western	1	1	100

© 2024 Cai J et al. *JAMA Network Open.*

National Clinical Research Center for Blood Diseases, Institute of Hematology & Blood Diseases Hospital, Chinese Academy of Medical Sciences & Peking Union	Tianjin	Eastern	6	6	100
First Hospital Affiliated to Zhengzhou University	Zhengzhou	Central	2	2	100
Chongqing Medical University Affiliated Children's Hospital	Chongqing	Western	5	5	100
**Total**			**112**	**101**	**90.2**

© 2024 Cai J et al. *JAMA Network Open.*

eTable 4. Demographic and Characters of Caregiver Respondents and Survivors (n=164).

**Age of survivors (years) Median (range)**		9.9 (4.6- 21.7)
**Sex of survivors**		
	Male	96(58.5)
	Female	68(41.5)
**Diagnosis**		
	Leukemia	67(40.9)
	Lymphoma or solid tumors	47(28.7)
	Underwent HSCT due to hematologic malignancies or metabolic diseases	50(30.5)
**Years post-therapy**		
	2-5 years	81(49.4)
	≥ 5 years	83(50.6)
**Hematopoietic stem cell transplantation**		
	Yes	61(37.2)
**Radiation therapy**		
	Yes	15(9.1)
**Relationship with survivors**		
	Parents	162(98.8)
	Grandparents	2(1.2)
**Level of education (caregiver)**		
	Primary school (1 - 5/6 grades)	22(13.4)
	Secondary school (6/7 - 9 grades)	49(29.9)
	High school or completed high school	25(15.2)
	University degree or above	68(41.5)
**Employment status**		
	Unemployed	48(29.3)
**Residence**		
	Rural	52(31.7)
	Urban	112(68.3)
**Type of health insurance**		
	Government-based/social insurance	136(82.9)
	Private insurance	6(3.7)
	No insurance	22(13.4)
**Annual income of the household (CNY*)**		
	<50,000	59(36.0)
	50,001-100,000	50(30.5)
	100,001-150,000	23(14.0)
	150,001-250,000	15(9.1)
	>250,000	17(10.4)

*1CNY≈0.138USD

HSCT: hematopoietic stem cell transplantation

© 2024 Cai J et al. *JAMA Network Open.*
