# Supplementary material for: Accessibility of and Barriers to Long-Term Follow-Up Care for Childhood Cancer Survivors
Source: JAMA Netw Open. 2024 Oct 17;7(10):e2440258. doi: 10.1001/jamanetworkopen.2024.40258 (PMC11581527; doi:10.1001/jamanetworkopen.2024.40258)
Supplement: Supplement 2. — Data Sharing Statement [file jamanetwopen-e2440258-s002.pdf]

Data Sharing Statement

Cai. Accessibility of and Barriers to Long-Term Follow-Up Care for Childhood Cancer Survivors. *JAMA Netw Open*. Published October 17, 2024.
doi:10.1001/jamanetworkopen.2024.40258

Data
Data available: Yes
Data types: Deidentified participant data
How to access data: caijiaoyang@scmc.com.cn
When available: With publication

Supporting Documents
Document types: None

Additional Information
Who can access the data: Investigators whose proposed use of the data has been approved by an independent review committee
Types of analyses: For individual participant data meta-analysis
Mechanisms of data availability: Proposals should be directed to Dr. Jiaoyang Cai (caijiaoyang@scmc.com.cn) after approval of a proposal and with a signed data access agreement
